# *European Journal of Heart Failure* expert consensus statement on transcatheter treatment of mitral regurgitation in heart failure

**DOI:** 10.1002/ejhf.70043

**Published:** 2025-10-30

**Authors:** Christos Iliadis, Marianna Adamo, Volker Rudolph, Nicole Karam, Ralph Stephan von Bardeleben, Stefan D. Anker, Victoria Delgado, Jörg Hausleiter, Hannah Kempton, Marco Metra, Michael Böhm, Stephan Baldus

**Affiliations:** ^1^ Department of Internal Medicine III Faculty of Medicine and University Hospital Cologne, University of Cologne, and Center for Cardiovascular Medicine (CCM^ABCD^), partner site Cologne Cologne Germany; ^2^ Cardiology, ASST Spedali Civili, Department of Medical and Surgical Specialties, Radiological Sciences, and Public Health University of Brescia Brescia Italy; ^3^ General and Interventional Cardiology, Heart and Diabetes Centre NRW University Hospital of the Ruhr‐University Bochum, Medical Faculty OWL Bad Oeynhausen Germany; ^4^ Department of Cardiology, European Hospital Georges Pompidou and Paris Cardiovascular Research Centre (INSERM U970) Paris France; ^5^ Department of Cardiology University Medical Centre Mainz Mainz Germany; ^6^ Department of Cardiology (CVK) of German Heart Centre Charité; German Centre for Cardiovascular Research (DZHK) partner site Berlin Charité Universitätsmedizin Berlin Germany; ^7^ Cardiovascular Imaging Section, Department of Cardiology Heart Institute, University Hospital Germans Trias i Pujol Badalona Spain; ^8^ Medizinische Klinik und Poliklinik I, LMU‐Klinikum, LMU München Munich Germany; ^9^ German Centre for Cardiovascular Research (DZHK), partner site Munich Heart Alliance Munich Germany; ^10^ Department of Cardiology, Angiology and Internal Intensive Care University Hospital of Saarland, Saarland University Homburg Germany

**Keywords:** Consensus statement, Heart failure, Mitral valve, Secondary mitral regurgitation, Transcatheter edge‐to‐edge repair

## Abstract

Secondary mitral regurgitation (SMR) is highly prevalent in patients with heart failure (HF), associated with poor prognosis, and its treatment is typically preceded by implementing pharmacotherapy for HF as well as cardiac resynchronization therapy. Given the increase in experience and important technological iterations, transcatheter mitral valve repair has witnessed increasing safety and efficacy. Recently, two randomized controlled trials extended the level of evidence for this intervention in patients with SMR: RESHAPE‐HF2 randomized patients with less severe SMR to either medical therapy alone versus adjunct transcatheter repair, whereas the MATTERHORN trial compared surgical therapy with transcatheter mitral valve repair in patients with SMR and increased risk for surgery. These two trials have a potential impact on the indications of transcatheter repair. Here we discuss updated indications for transcatheter mitral valve therapy across the different subtypes of SMR, revisit the current body of evidence for transcatheter mitral valve repair and classify this technique into the current treatment hierarchy of SMR in patients with HF.

## Introduction

Secondary mitral regurgitation (SMR) is highly prevalent in patients with heart failure (HF) and associated with poor prognosis. While the final common pathway is dysfunction of the mitral valve, the aetiology of SMR is heterogeneous, and can include any or a combination of left ventricular (LV) remodelling and/or isolated annular dilatation, driven by either atrial fibrillation (AF), atrial myopathy and/or LV diastolic dysfunction.

The algorithms for therapy follow the current guidelines of treatment of HF with guideline‐directed medical therapy (GDMT) being the principal pillar. However, in addition to GDMT, mitral valve transcatheter edge‐to‐edge repair (M‐TEER), and to a lesser extent surgical repair or replacement, have gained importance due to accumulating evidence of symptomatic improvement, reduced HF hospitalization (HFH) and, in some cases, also death.

Based on the outcomes of two large randomized trials (COAPT and RESHAPE‐HF2), the new 2025 European Guidelines have updated their recommendations for intervention in SMR. In light of current data evaluating transcatheter therapy in combination with GDMT and in comparison with mitral valve surgery, this expert consensus revisits the current recommendations for treatment in patients with HF and SMR.

## Pathophysiology

Secondary mitral regurgitation is a complex disease, resulting from an imbalance between increased tethering forces (due to global and/or focal LV dilatation, papillary muscle displacement and/or dysfunction) and decreased closing forces (reduced LV contractility and/or synchronicity) in the presence of a structurally normal mitral valve. Typically, SMR emerges as a chronic disease resulting from progressive remodelling of the left chambers. Acute mitral regurgitation (MR) results from instantaneous disintegration of different constituents of the mitral valve apparatus, which is not included in SMR. For example, ruptured papillary muscle secondary to myocardial infarction is classified as primary ischaemic MR. Carpentier's classification highlights two principal mechanisms of leaflet motion in SMR: type I, with normal leaflet motion due to annular dilatation from the adjacent chambers (left ventricle and/or left atrium); and type IIIb, characterized by restricted leaflet motion due to global or regional LV/left atrial (LA) remodelling. Rarely, such as in chronic ischaemic SMR, fibrosis and elongation of the papillary muscle yielding leaflet prolapse can provoke MR which resembles type II disease according to Carpentier's classification, despite the fact that the disease is originating in the ventricle.^1^ Annular dilatation provokes tethering and reduced annular dynamics hamper adequate mitral valve closing, leading to SMR. Further, in more advanced stages of LV and/or (LA) dilatation, tethering becomes more pronounced in the subvalvular region.^2^ Dilated cardiomyopathy causes leaflet tethering due to mainly symmetric enlargement of the left ventricle. In ischaemic cardiomyopathy, MR can be a result of either anterior myocardial infarction causing papillary displacement or apical tethering. Conversely, posterior wall infarction is associated with apical displacement of the posterior papillary muscle provoking asymmetric tethering with a larger posterior leaflet angle,^3^, ^4^ causing insufficient closure of the mitral valve. Both are hallmarks of ventricular SMR (vSMR). In contrast, atrial SMR (aSMR) is the result of higher tethering forces due to LA dilatation and impaired annular dynamics resulting from increased LA pressure and atrial dysfunction as observed in HF with preserved ejection fraction (HFpEF) and/or AF.^5^ Atriogenic leaflet tethering is a complex pathoanatomic subtype of aSMR disease, which is based on marked LA dilatation posteriorly, and thus bending of the posterior leaflet over the LV crest, leading to reduced coaptation and typically eccentric MR.^6^ Importantly, this subgroup of aSMR is associated with worse procedural and clinical outcomes after M‐TEER.^7^

## Epidemiology and prognosis

The prevalence of moderate or severe MR is high in the community, affecting about 0.6% of all adults.^8^ Of these, 65% are diagnosed for SMR, being the most frequent MR aetiology.^9^ Within this patient population, SMR was characterized as vSMR driven in 58% and as aSMR in 42%. Patients with vSMR were slightly younger than patients with aSMR (73 ± 14 years vs. 80 ± 10 years). Female sex was predominant in aSMR (67%), whereas male sex was slightly more prevalent in vSMR (59%). Patients diagnosed for vSMR share similar characteristics as patients with HF with reduced ejection fraction (HFrEF) based on atherosclerotic disease; in contrast, patients with aSMR are typically diagnosed for HFpEF and/or AF. In patients hospitalized for acute HF, the prevalence of MR is reported to be between 24% and 53% in different registries and associated with worse outcomes.^10^

Overall, patients with SMR have dismal prognosis.^9^ Irrespective of the underlying myocardial disease, SMR has been shown to impact on outcomes, independent of other variables, including those related with severity of HF, pulmonary hypertension, right ventricular dysfunction, tricuspid regurgitation and chronic obstructive pulmonary disease.^9^, ^11^, ^12^, ^13^, ^14^, ^15^ Patients with SMR experience an excess risk of mortality during a follow‐up time of 4.6 ± 3.1 years: patients with vSMR revealed the highest mortality, with a risk ratio of 3.45 (2.98–3.99) whereas patients with aSMR demonstrated a slightly better prognosis (risk ratio 1.88; 1.52–2.25). Further, the risk for progression of HF is considerable, reaching 83 ± 3% in vSMR and 59 ± 4% in aSMR over 5 years, respectively.^9^

## Diagnosis of secondary mitral regurgitation

Clinical examination of SMR is often difficult, as it is the consequence of initial ventricular/annular disease, which are present long before SMR develops. Thus, symptom severity may not correlate with SMR severity. Further, as patients with chronic HF may present with acute deterioration and fluid overload, measures to optimize decongestion are mandated to avoid overestimation of SMR severity in the acute phase. Detailed assessment of SMR by imaging has the objective of defining the underlying aetiology (ventricular vs. atrial), the regurgitation grade, suitability for transcatheter intervention and concomitant insults of the ventricles and the pulmonary circulation. Transthoracic echocardiography (TTE) is the principal imaging technique to assess the majority of the above indicated pathoanatomic characteristics. Key differences have been highlighted in the section on pathophysiology above. Both types of SMR can display varying grades of mitral annulus calcification which may pose therapeutic challenges, particularly if the calcification extends to the mitral leaflets. The anatomic characteristics (extent of tethering, tenting angle, coaptation gap, leaflet length, jet eccentricity and localization, as well as the dilatation of the mitral valve annulus) are better appreciated using transoesophageal echocardiography (TOE), with three‐dimensional data being a prerequisite for assessing the suitability of (transcatheter) repair regarding mitral valve orifice and calcifications in each scallop in detail. Moreover, three‐dimensional TOE allows the display of multiplanar reconstructions of the mitral valve and the identification of the precise site of disease and origination of the jet.

Assessment of the MR grade using echocardiographic techniques requires a multiparametric approach evaluating qualitative, semi‐quantitative and quantitative parameters (*Table* *1*). When four or more of the qualitative and semi‐quantitative parameters coincide, in the presence of LV dilatation and dysfunction and LA dilatation, MR can be graded as ‘severe’. However, for complete assessment, current recommendations advocate for quantification of the effective regurgitant orifice area (EROA), the regurgitant volume and the regurgitant fraction. The EROA and the regurgitant volume are usually measured with the two‐dimensional proximal isovelocity surface area (PISA). This method has several limitations that may lead to underestimation of MR severity. First, the PISA method assumes that the regurgitant orifice is circular which is not common in SMR. Usually, the anatomical regurgitant orifice is slit‐like or oval. Colour Doppler three‐dimensional echocardiography allows for multiplanar reconstructions of the regurgitant orifice and the planimetry of the anatomical regurgitant orifice area. This measurement has shown to provide a better estimation of the severity of MR than two‐dimensional echocardiography, using as reference cardiac magnetic resonance. In addition, low‐flow conditions in patients with SMR facilitate underestimation of the regurgitant volume and therefore, SMR should be assessed cautiously in particular during a state of low loading conditions. Moreover, assessment of regurgitant volume only considers the measurement of the convergence zone (PISA radius) in a single systolic frame despite its temporal changes, namely being maximal at the beginning and the end of the systole. The measurement of the regurgitant fraction may overcome this limitation in the absence of concomitant aortic regurgitation. The regurgitant fraction is calculated as the difference between the LV stroke volume and the forward stroke volume (measured at the LV outflow tract with pulsed‐wave Doppler) relative to the LV stroke volume. The LV stroke volume can be measured as the difference between the end‐diastolic and end‐systolic volumes or based on pulsed‐wave Doppler measurements of the mitral inflow, leading to discrepant results. Current guidelines consider MR severe when the EROA is ≥0.4 cm^2^, the regurgitant volume is ≥60 ml, and the regurgitant fraction is ≥50%. In SMR specifically, considering the limitations of this quantification technique and the load dependency of SMR, EROA ≥0.3 cm^2^ and/or a regurgitant volume ≥45 ml can define severe MR.^1^ Additional techniques based on colour Doppler three‐dimensional TOE have been developed to quantify the regurgitant volume along the systole similarly to cardiac magnetic resonance. Based on algorithms that track the convergence zone of the regurgitant jet, the regurgitant volume is displayed as a function of time.^16^

**Table 1:** Qualitative, semi‐quantitative, and quantitative parameters of severe secondary mitral regurgitation on transthoracic echocardiography

Qualitative	Large coaptation defect of the mitral leaflets Severe tenting Large regurgitant central jet occupying >50% of the left atrial area Eccentric jet with Coanda effect (swirling along the atrial wall) Large holosystolic convergence zone
Semi‐quantitative	Vena contracta width of the regurgitant jet of ≥7 mm (or ≥8 mm in the bicommissural view) Pulmonary vein systolic flow reversal on pulsed‐wave acquisitions Velocity–time integral of the mitral valve relative to the that of the left ventricular outflow tract of >1.4 E‐wave dominant (1.2 m/s)
Quantitative	Effective regurgitant orifice area ≥0.4 cm^2^ (or ≥0.3 cm^2^ if elliptical orifice) Regurgitant volume ≥60 ml (or ≥45 ml if low‐flow) Regurgitant fraction ≥50%

When the symptoms of the patient do not match the severity of SMR on echocardiography at rest, exercise echocardiography can be useful and is recommended in unmasking severe SMR.^17^ The increase in EROA during peak exercise is associated with poor outcomes.^18^ Furthermore, when the echocardiographic assessment of SMR severity is inconclusive, cardiac magnetic resonance is the method of choice to quantify LV and LA volumes and regurgitant volume and fraction. There are several techniques to evaluate the regurgitant volume and fraction but the most frequently used are the standard method that calculates the stroke volume on cine images and the flow through the aorta with phase contrast images as well as four‐dimensional flow that reconstructs the three‐dimensional mitral regurgitant jet.^19^ Furthermore, cardiac magnetic resonance has the unique characteristic of providing the tissue characterization of the myocardium. In patients with SMR, the presence of myocardial scar and fibrosis has been associated with worse outcomes and less functional improvement after mitral valve intervention.^20^, ^21^

## Treatment algorithm in patients with heart failure and secondary mitral regurgitation

A holistic therapeutic approach is required when dealing with patients with SMR and HF. In patients with HFrEF, optimization of medical treatment has to be achieved, or at least targeted, to the maximum tolerated individual patient's level. The goal is to reduce mortality and morbidity, but also to reduce the severity of SMR and thereby halt progression of ventricular disease. Indeed, among patients with SMR, all four mortality‐modifying drug classes (angiotensin‐converting enzyme inhibitors/angiotensin receptor blockers, and angiotensin receptor–neprilysin inhibitors; beta‐blockers; mineralocorticoid receptor antagonists; and sodium–glucose co‐transporter 2 inhibitors) have been shown to be associated with SMR reduction.^22^, ^23^, ^24^, ^25^, ^26^, ^27^, ^28^, ^29^ In acute HF and MR, vasodilators are first line‐therapy to reduce LV afterload in absence of hypotension, increasing forward stroke volume and reducing MR. Otherwise, inotropic (dobutamine) and inodilator drugs (milrinone) may be appropriate in patients with hypotension or hypoperfusion, whereas vasopressors may worsen MR and should be used at the lowest possible dose. Intravenous diuretics should be titrated until adequate decongestion is achieved.^10^

Cardiac resynchronization therapy (CRT) is endorsed as a class I recommendation for patients with LV ejection fraction (LVEF) ≤35%, a QRS duration ≥130 ms and left bundle branch block morphology who remain symptomatic despite optimal medical treatment both for symptomatic improvement and reduction in HFH and mortality. Interestingly, in CARE‐HF those patients with moderate to severe MR, defined by a MR area ratio ≥0.218, had a numerically larger reduction in the composite of death from any cause or an unplanned HFH for a major cardiovascular event with CRT, compared with those with less severe MR^30^—further illustrating the prognostic relevance of SMR. MR reduction appears to be driven by instant effects of CRT on asynchrony, rather than by long‐term LV reverse remodelling.^31^ Beneficial effects on MR reduction by CRT have been shown in several subsequent studies, further underlying the importance of CRT in this subpopulation.^32^, ^33^ Of note, baseline moderate to severe SMR that remained unchanged at 6 months after CRT was independently associated with increased risk of mortality (hazard ratio [HR] 1.77; 95% confidence interval [CI] 1.41–2.22, *p* < 0.001).^34^ Restoration of sinus rhythm, through pharmacological measures, cardioversion and ultimately catheter ablation has also been shown to reduce SMR, especially in patients with aSMR.^35^, ^36^

In patients with persistent SMR and HF symptoms despite maximal tolerated GDMT including CRT if indicated, mitral valve repair, in particular M‐TEER, should be considered the treatment of choice. However, also in the context of transcatheter therapy, careful risk stratification and appropriate patient selection is warranted, and several predictors indicating prognosis have been identified. Among those, pulmonary hypertension and right ventricular dysfunction should be systematically assessed, since they represent the most robust parameters to identify non‐responders to M‐TEER therapy.^37^, ^38^, ^39^, ^40^ Conversely, the proportionality concept stratified SMR according to LV dimensions: disproportionate SMR, that is, severe SMR as assessed by EROA in relation to LV end‐diastolic dimension, appears to represent a pathology, which identifies patients who reveal the most prognostic benefit. However, this concept remains controversial given that the benefit of M‐TEER in the COAPT trial did not fully support this hypothesis in a post‐hoc analysis.^41^ Recently, an artificial intelligence‐derived risk score, the EuroSMR risk score, has been developed for the prediction of mortality and futility using clinical, echocardiographic, laboratory and data of pharmacological HF interventions.^42^ The most predictive parameters were N‐terminal pro‐B‐type natriuretic peptide (NT‐proBNP), haemoglobin, right atrial area, New York Heart Association (NYHA) class, systolic pulmonary artery pressure, and age. Developed specifically for the SMR population, the score was superior in predicting 1‐year mortality after M‐TEER when compared with EuroScore II, MitraScore, and the COAPT risk score.

Several studies, including randomized controlled trials and registries, revealed that patients treated with M‐TEER are often on suboptimal GDMT prior to the procedure, in large part due to hypotension. However, a significant fraction (38%) of patients experiences haemodynamic improvement after M‐TEER, which subsequently allows for initiation of a new HF drug class and/or up‐titration of at least one drug class, which translates into lower rates for mortality and HFH.^43^ Accordingly, further optimization of GDMT should be targeted after M‐TEER, which supports the potential catalytic role of M‐TEER in optimizing medical treatment of HFrEF. Improved haemodynamics after MR treatment seems not to be mediated by LV reverse remodelling, according to a post‐hoc analysis of the COAPT trial, which contradicts findings from observational studies.^44^

## Current transcatheter devices for treatment of secondary mitral regurgitation

The development and clinical implementation of M‐TEER, the transcatheter approach of approximation of anterior and posterior leaflet of the mitral valve using a clip‐like system, has transformed the management of MR, and in particular SMR. M‐TEER provides a minimally invasive therapeutic option for high‐risk surgical candidates. The MitraClip (Abbott, Abbott Park, IL, USA) and PASCAL (Edwards Lifesciences, Irvine, CA, USA) are the two current commercially available M‐TEER systems. After continuous iteration, both systems are now offered in multiple device sizes, in order to serve the wide range of anatomies.

The anatomic suitability criteria for procedural success for M‐TEER include the achievement of favourable transseptal access to the left atrium, the absence of calcification in the treatment zone of the leaflets, adequate posterior mitral leaflet length (>10 mm), and the absence of perforations, significant clefts or a preexisting mitral stenosis (*Table* *2*).^45^ In fact, M‐TEER treatment zone and the number of lesions to be treated are factors to be considered for the risk of mitral stenosis besides the mitral valve orifice area.^46^ The aetiology of vSMR did not affect outcomes after M‐TEER in the COAPT trial.^47^ Given the risk of residual MR or MR recurrence following this type of mitral valve repair, we should consider important factors associated with higher residual or recurrent MR in patients screened for M‐TEER. In vSMR specifically, LA volume has been identified as a risk factor for MR recurrence.^48^ Further, a low leaflet‐to‐annulus index proved to be a significant predictor of higher residual MR after M‐TEER.^49^ In SMR, LA volume and a low leaflet‐to‐annulus index were similarly predictive of higher residual MR after M‐TEER.^50^

**Table 2:** Criteria for mitral valve transcatheter edge‐to‐edge repair treatment success in secondary mitral regurgitation

M‐TEER complexity	Non‐complex	Complex	Very complex	Not feasible
M‐TEER eligibility	Optimal	Suitable	Limited	Not recommended–consider replacement
Localization of pathology	A2‐P2	Commissural (A1‐P1 or A3‐P3)	Over all mitral valve scallops	Over all mitral valve scallops
Mitral valve orifice area	>4 cm^2^	3.5–4.0 cm^2^	3.0–3.5 cm^2^	<3.0 cm^2^
PML length	>10 mm	7–10 mm	5–7 mm	<5 mm
Tenting height	<10 mm	>10 mm	>10 mm	>10 mm
Regurgitation jet extent	One isolated jet	Max 2 jets	Very wide/jet extent over all mitral valve scallops	Very wide/jet extent over all mitral valve scallops

M‐TEER, mitral valve transcatheter edge‐to‐edge repair; PML, posterior mitral leaflet.

In contrast to M‐TEER, transcatheter mitral valve replacement (TMVR) technologies for the treatment of SMR have emerged late. Until very recently, the only CE‐marked bioprosthetic device, the Tendyne valve (Abbott Structural Heart, Santa Clara, CA, USA) was introduced into the market in 2020, requiring transapical minimal invasive surgical access and fixation of an apical tether. A second valve, the Sapien M3 (Edwards Lifesciences) has most lately received CE‐mark, being the first approved transseptal device. Data from the ENCIRCLE trial (NCT04153292) are expected to be published soon. The paucity of TMVR devices is in part due to the complex anatomy of the mitral valve, requiring a valve that tethers inside the mitral valve annulus without obstructing the LV outflow tract. M‐TEER is safe and efficacious, with 30‐day mortality rates below 5%.^51^, ^52^ In contrast, transapical TMVR has a 30‐day mortality rate of up to 9%,^53^ and therefore remains reserved for patients at increased risk for surgery and valvular anatomy not suitable for M‐TEER.

Additional TMVR devices in current clinical trials include the Medtronic Intrepid valve, which has now achieved trial approval in the USA, Canada and EU, re‐designed as a new 29 F transseptal implant device that shows promising preliminary safety and efficacy data but is not yet subject of a randomized trial. Additional transseptal replacement devices include the CardioValve, and the AltaValve, the latter of which may overcome LV outflow tract obstruction, however, is dependent on atrial chamber fixation. Highlife may receive CE‐mark in the near future and involves a combination of a mitral chordal and leaflet docking device and the implant of a transseptal conventional transcatheter aortic valve‐like implant that seals within the docking implant. Most TMVR strategies still have high screen failure rates of 50%–80% due to size mismatch with annular diameters or the risk of LV outflow tract obstruction. Thus, in future, the benefits of TMVR, with the potential to achieve almost complete resolution of MR will have to be balanced against the exceptionally high safety profile of M‐TEER, and randomized comparisons are needed to evaluate the differences between both approaches with respect to quality of life, HFH and mortality.

## Current guideline recommendations and criteria for prognostic benefit

In the current HF and previous valvular heart disease guidelines, M‐TEER received a class IIa level B recommendation for patients with significant secondary MR, which remained symptomatic despite optimized GDMT, and fulfilling the criteria that define a high probability of having a favourable response to the procedure (COAPT criteria).^54^, ^55^, ^56^ The current VHD guidelines raised the level of recommendation for M‐TEER to class I. This is based not only on the long term outcome of patients in COAPT, but importantly on the results of the RESHAPE HF 2 trial.^57^

RESHAPE‐HF2 enrolled 505 patients with HFrEF and moderate‐severe or severe SMR (EROA ≥0.2 mm^2^),^58^ randomized 1:1 to receive optimized GDMT alone versus M‐TEER in addition to GDMT. The trial included three prespecified primary endpoints: cardiovascular death or HFH, HFH (both assessed after 2 years), and quality of life measured with the Kansas City Cardiomyopathy Questionnaire (KCCQ) at 1 year, respectively. At 2‐year follow‐up, there was a significant reduction in cardiovascular death or HFH (HR 0.64; 95% CI 0.48–0.85, *p* = 0.002), HFH (HR 0.59; 95% CI 0.42–0.82, *p* = 0.002). At 1‐year follow‐up, quality of life was improved with a KCCQ overall summary score mean difference of 10.9 points (95% CI 6.8–15.0, *p* < 0.001). The reduction in HFH drove the primary endpoint, whereas all‐cause mortality was numerically reduced but not statistically significant; this was potentially blunted by cross‐over of 38 patients (15%) from the control arm in the first 2 years. In the study level meta‐analysis published simultaneously with RESHAPE‐HF2, which merges the primary results of RESHAPE‐HF2, COAPT and MITRA‐FR, a clear benefit of M‐TEER in terms of 2‐year HFH and a trend towards a mortality benefit of M‐TEER was observed.^59^, ^60^ RESHAPE‐HF2, similarly to COAPT but conversely to MITRA‐FR, excluded patients with severe right ventricular failure and severe tricuspid regurgitation, as well as haemodynamically unstable patients.^61^ Patients enrolled into the three trials were comparable for age and systolic LV function. However, patients included in RESHAPE‐HF2 had a mean EROA of 0.23 cm^2^ (only 14% of patients had an EROA >0.40 cm^2^, and 23% had an EROA <0.20 cm^2^), whereas COAPT and MITRA‐FR patients were indicative of a mean EROA of 0.40 and 0.31 cm^2^, respectively (*Table* *3*). Furthermore, patients included in RESHAPE‐HF2 compared with those included in the latter studies, revealed lower levels of NT‐proBNP, higher glomerular filtration rates, and more frequently received optimized GDMT^62^: in RESHAPE‐HF2, 96% of patients received beta‐blocker therapy (vs. 90% in COAPT and 90% in MITRA‐FR), 82% were treated with mineralocorticoid receptor antagonists (vs. 50% in COAPT and 55% in MITRA‐FR), 82% with renin–angiotensin system inhibitors (vs. 67% in COAPT and 84% in MITRA‐FR).

**Table 3:** Comparison of echocardiographic characteristics in mitral valve transcatheter edge‐to‐edge repair trials

	COAPT	MITRA‐FR	RESHAPE‐HF2	EXPANDed (SMR cohort)		MiCLASP (SMR cohort)		MATTERHORN
				SMR 2+	SMR 3+	SMR 2+	SMR 3+	
LVEF (%)	31	33	32	40	39	44	39	43
LVEDV (ml)	192	252	205	164	174	156	196	165
EROA (cm^2^)	0.41	0.31	0.23	0.20	0.30	0.22	0.36	0.22
PASP (mmHg)	44	54	41	46	49	40	46	39 + CVP

CVP, central venous pressure; EROA, effective regurgitant orifice area; LVEDV, left ventricular end‐diastolic volume; LVEF, left ventricular ejection fraction; PASP, pulmonary arterial systolic pressure; SMR, secondary mitral regurgitation.

The robust adherence to HF medication in the RESHAPE‐HF2 population may further add to the blunted effect on mortality by M‐TEER as compared with COAPT. By demonstrating the effectiveness of M‐TEER even in patients with less severe MR, RESHAPE‐HF2 may however indicate a state of disease in which M‐TEER has also the potential to improve outcome at an earlier stage of disease, in particular in absence of severe right ventricular dysfunction and/or tricuspid regurgitation.^37^, ^63^ At the same time, a prespecified analysis of RESHAPE‐HF2^58^, ^64^ revealed that study participants who required HFH in the year prior to randomization derived greater prognostic benefit from M‐TEER than those without HFH prior to randomization (*p* for interaction = 0.03 for the composite of cardiovascular death and HFH at 2 years), illustrating the potential prognostic benefit of this therapy in those with subtle indices of disease progression.

Additional evidence for the benefit of M‐TEER in patients with symptomatic but moderate SMR derives from sub‐analyses of the MiCLASP and EXPANDed prospective studies: results from the MiCLASP study reported sustained MR reduction, as well as improved symptoms, functional status and hospitalization rates.^65^ These results were echoed in the outcomes from the EXPANDed trial.^66^ Importantly, both studies confirmed similar outcomes for mortality and HFH as well as patient‐centred outcomes including NYHA class and KCCQ improvement after M‐TEER in patients with ‘moderate’ or severe MR. It is important however to stress the limitations of assessing improvement in quality of life in unblinded observational studies. To reconcile the outcome of these trials, the ‘disproportionate MR’ theory has been revisited to place more emphasis on LV dilatation as a determinant of outcomes.^67^ Considering the complex aetiology of SMR, it would seem more appropriate to contextualize SMR to the degree of LV dilatation, reflecting the stage of disease with more precision.

Highlighting the significance of the stages of cardiac failure on outcomes after M‐TEER, results of a recent analysis from the EuroSMR registry demonstrated a prognostic impact on mortality and symptomatic improvement after M‐TEER, based on the stages of cardiac failure, with the worst outcomes observed in patients with established LV and right ventricular failure.^39^ Adding to this is the observation that pulmonary pressures in MITRA‐FR were significantly higher than those measured in the COAPT, RESHAPE‐HF2, EXPANDed, or MiCLASP cohorts (*Table* *3*). This is in line with higher LV end‐diastolic volumes in MITRA‐FR, which may be a determinant for the lack of benefit for M‐TEER in this trial.

Whereas the stage and severity of LV failure are critical considerations in SMR cohorts, attention should also be given to the criteria used to define severe SMR. In most real‐world settings, including the RESHAPE‐HF2, EXPANDed, and MiCLASP studies, SMR severity is assessed in the clinical context: patients were characterized with respect to clinical symptoms, a history of HF, laboratory values (e.g. NT‐proBNP), followed by TTE and TOE and finally right heart catheterizations (e.g. v‐wave of the pulmonary capillary wedge pressure). The core‐lab readings of SMR severity in these studies were performed after study inclusion and based on the TTE studies. This is in contrast to trials like COAPT, in which core‐lab based TTE assessment pre‐selected patients prior to inclusion, resulting in a SMR subcohort of patients with higher EROA and regurgitant volume measurements. Quantification of EROA using the PISA method has well‐recognized limitations. As a two‐dimensional measurement, the PISA method fails to capture the true three‐dimensional complexity and volume of SMR. This raises the important question of whether the ‘moderate’ MR reported in RESHAPE‐HF2, EXPANDed, and MiCLASP was truly moderate, or in fact underestimated due to methodological limitations of EROA. To illustrate this, stroke volume estimates were extrapolated from EROA values in ‘COAPT‐like’ patients (i.e. with LVEF 35%, LV end‐diastolic volume 200 ml and heart rate 75 bpm), and shown to be unfeasible, suggesting that the current EROA and regurgitant volume thresholds for classifying severe SMR in HFrEF patients by TTE might need to be revisited.^45^ Additionally, findings from over 1800 patients in the EuroSMR registry showed no correlation between EROA and mortality, challenging the utility of EROA as a prognostic metric in SMR.^68^

**Figure 1:**
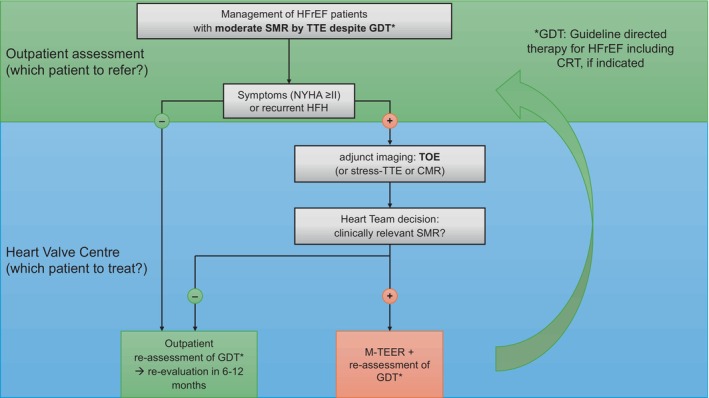
Proposed management of heart failure with reduced ejection fraction (HFrEF) patients with moderate secondary mitral regurgitation (SMR). CMR, cardiac magnetic resonance; CRT, cardiac resynchronization therapy; GDT, guideline‐directed therapy; HFH, heart failure hospitalization; M‐TEER, mitral valve transcatheter edge‐to‐edge repair; NYHA, New York Heart Association; TOE, transoesophageal echocardiography; TTE, transthoracic echocardiography.

Taken together, discrepancies among M‐TEER HF trials may be attributed to differences in disease stage and HF severity. In addition, caution is warranted when interpreting these trial data based solely on classifications of ‘moderate’ MR, as current EROA thresholds may not accurately reflect true MR severity, particularly using only TTE. Therefore, when assessing the suitability for M‐TEER, a more holistic approach is advisable and should replace the reliance on a single two‐dimensional echocardiographic parameter: this should include clinical symptoms, LV size and function, markers of HF progression (such as LA function and pulmonary hypertension), and adherence to and tolerance of GDMT. *Figure* *1* summarizes a proposed management of HFrEF patients with moderate SMR.

Thus, RESHAPE‐HF2 added important evidence for M‐TEER in patients with HF and concomitant MR; however, rather than interpreting these results as evidence for a benefit of M‐TEER in all patients with MR 2+, results should be contextualized to LV function, and interpreted with an understanding of the limitations of the EROA. *Table* *4* summarizes relevant limitations of SMR trials.

**Table 4:** Limitations of secondary mitral regurgitation trials

No RCT comparing M‐TEER vs. OMT in aSMR No RCT comparing TMVR vs. OMT or vs. M‐TEER EROA quantification as primary criterion for SMR severity is challenging No clear benefit of M‐TEER in all patients with moderate SMR Patients with advanced HF or acute HF not included in trials Differences between SMR trials not explained through theoretical proportionality framework

EROA, effective regurgitant orifice area; HF, heart failure; M‐TEER, mitral valve transcatheter edge‐to‐edge repair; OMT, optimal medical therapy; RCT, randomized controlled trial; SMR, secondary mitral regurgitation; TMVR, transcatheter mitral valve replacement.

## Mitral valve transcatheter edge‐to‐edge repair versus mitral valve surgery

The current European guidelines assign a class IIb level C recommendation to isolated mitral valve surgery and a class I level B recommendation to concomitant valve surgery in patients with SMR undergoing coronary artery bypass grafting or other cardiac surgery. At the same time, surgery for severe aSMR received a class IIa level B recommendation.^57^

With regard to surgical techniques, the initially propagated restrictive annuloplasty failed to show superiority over valve replacement and resulted in MR recurrence rates of 33% and 59% after 1 and 2 years of follow‐up, respectively.^69^, ^70^ Nevertheless, post‐hoc‐analyses showed that patients with durable repair had a greater degree of reverse remodelling as compared with those having received mitral valve replacement.^70^ Whereas a selection bias may contribute to this observation, more recent data on adjunctive subvalvular surgical techniques lend support to further pursuing valvular repair in SMR. In particular, papillary repositioning and re‐alignment in addition to annuloplasty potentially yields lower MR recurrence rates and more pronounced reverse LV remodelling as reported in a small single‐centre randomized trial and in observational studies.^71^, ^72^

The current 2025 ESC guidelines however increased the level of recommendation for M‐TEER over surgery for isolated SMR. This is in large part based on the results of the MATTERHORN trial. The multicentre MATTERHORN trial randomized patients with isolated SMR to M‐TEER versus mitral valve surgery, with the choice between repair or replacement, as well as the addition of concomitant surgical procedures such as ablation at the discretion of the surgeon.^73^ A total of 210 symptomatic HF patients were enrolled. Patients were on GDMT and at increased risk but suitable for surgery. The cohort differed substantially from other randomized M‐TEER trials, with a mean LVEF as high as 43 ± 12% and annular dilatation rather than ventricular tethering as the predominant mechanism of SMR in approximately half of the patients. In the surgical group, valve repair was performed in 72% and replacement in 28%. The primary endpoint of death, HFH, MR recurrence, assist device implantation or stroke at 1 year occurred in 17% in the M‐TEER group and 23% in the surgical group, demonstrating non‐inferiority of M‐TEER versus surgery (*p* < 0.001). Recurrence of MR grade >2+ at 1 year was 2% in the surgical group and 9% in the M‐TEER group, which—despite the numerical difference—fulfilled prespecified criteria of non‐inferiority. The low MR recurrence rates in the surgical cohort may support the described recent advancements in MR repair techniques, however also reflect the high proportion of valve replacement in 28% of cases. The primary safety endpoint, a composite of major adverse events at 30 days, was reduced by an absolute difference of almost 40% in the M‐TEER group (15% vs. 55%, *p* < 0.001), thus fulfilling the prespecified criterion of non‐inferiority. This difference remained through 1 year, and after omitting bleeding and AF recurrence from the endpoint. A non‐prespecified post‐hoc analysis in patients with aSMR reproduced the overall results of MATTERHORN, supporting the new class IIb level B recommendation for M‐TEER in patients with this pathology.^74^ Clearly, longer‐term data will be of importance, particularly for younger patients without overt surgical risk.

Taken together, critical re‐appraisal of the role of surgery in SMR is warranted, in light of the paucity of evidence and the above‐described trial results for M‐TEER. This will extend to the evaluation of surgery in patients with additional valvular disease, in whom additional transcatheter valvular therapies can now be offered, or patients with concomitant coronary disease, in whom the effect of revascularization on LV remodelling still remains uncertain.

## Open questions

### Timing of mitral valve transcatheter edge‐to‐edge repair

There are many open questions about the correct sequence for implementing available medical and interventional treatment options. While GDMT is unquestionably the backbone of HF therapy, SMR patients with advanced HF are typically excluded or severely underrepresented in pharmacological trials for HF. Notably, dedicated trials in advanced HF, with presumably a high prevalence of SMR, provide equivocal results.^75^ Importantly, less than 39% of patients in the COAPT trial were able to tolerate more than two GDMT substance classes, with the predominant reason for intolerance being hypotension.^76^ Employing M‐TEER in such patients improved GDMT tolerability and facilitated up‐titration.^43^ Enabling GDMT by M‐TEER emerges as an intriguing concept, which should be evaluated further in future studies. Also, the event curves in COAPT early separated in favour of M‐TEER, indicating that in patients adequately pre‐treated and fulfilling the COAPT criteria, M‐TEER should not be withheld unnecessarily.

### Interaction with cardiac resynchronization therapy

Another open question relates to the timing of CRT in patients with SMR. Whereas CRT trials revealed some effect on MR grade, patients with truly severe SMR were underrepresented in CRT trials. Thus, while CRT is a valuable treatment option if otherwise indicated, its role in patients with severe SMR, who carry a high risk for acute or progressive HF, is less clear.^77^ As mentioned above, sub‐analyses of CARE‐HF show a greater benefit in patients with ≥ moderate MR. Whether earlier introduction of CRT would be associated with improved clinical outcomes by facilitating up‐titration of GDMT is currently under debate.^78^

### Interaction with interventions for atrial fibrillation

Catheter ablation of AF has recently been shown to confer a clinical benefit in patients with stable as well as advanced HF.^79^, ^80^ In the context of MR, only observational data are available, and the majority of patients in these studies exhibit non‐severe MR.^81^ Reductions in MR severity after AF treatment were modest.^82^ Whereas the existence of SMR is a predictor of AF recurrence after pulmonary vein isolation, the presence of AF does not blunt the beneficial treatment effects of M‐TEER.^83^ Whether the duration of AF or the extent of MR will ultimately help guide the sequence of the two interventions still remains to be determined. Data on the role of M‐TEER in stabilizing sinus rhythm also need to be generated.

### Mitral valve transcatheter edge‐to‐edge repair in acute heart failure

Acute HF complicated with MR is a challenging situation for patients and physicians and has many facets, encompassing acute HF with or without acute myocardial ischaemia. Data on M‐TEER therapy in acute settings remain scarce compared with chronic MR. The most life‐threatening condition resulting in severe acute MR is papillary muscle rupture after myocardial infarction, which requires immediate treatment. In patients with prohibitive surgical risk, M‐TEER may be appropriate in expert centres, considering the very high risk for adverse events in severe comorbidity.^10^ Registry data show significantly worse outcomes in this subset of patients, compared with MR following LV failure in the chronic phase after myocardial infarction.^84^ However, the optimal timing of intervention in the latter is unclear, and depends on the clinical course. Primary percutaneous coronary intervention and medical therapy potentially enables staged and not emergency M‐TEER.^10^ In a recent meta‐analysis, patients presenting with cardiogenic shock and MR without acute myocardial infarction had better 30‐day but worse intermediate‐term (mean 10.6 ± 6.4 months) outcomes despite similar acute device success as compared with patients with acute myocardial infarction.^85^ These data support the tenet that patients with MR upon acute‐on‐chronic HF complicated by cardiogenic shock have worse prognosis—irrespective of and despite successful therapy of the valvular lesion.

### Heterogeneity of secondary mitral regurgitation populations

As discussed above, randomized controlled trials investigating the role of M‐TEER in SMR used divergent measures for the definition of relevant MR and included patients with substantial differences in LV volumes.^47^, ^58^, ^59^ This raises the question of whether there is an upper LV volume at which M‐TEER may be too late. To that end, a framework relating the clinical benefit of M‐TEER to the degree of LV dilatation has recently been proposed, but requires further systematic investigation.^67^

Despite the significant effects of M‐TEER on mortality and HFH, the 5‐year follow‐up of the patients enrolled in COAPT showed a poor outcome with a 33% annualized rate of HFH, a 57% all‐cause mortality through 5 years, and a death or HFH rate of 74%.^86^ Thus, patients with severe SMR and HFrEF remain at high risk, and implementation of monitoring strategies and evidence‐based treatment remains of paramount importance and includes shared decision‐making along the patient's journey, when opting for mechanical circulatory support or transplantation or palliative care, respectively.

## Conclusions

**Figure 2:**
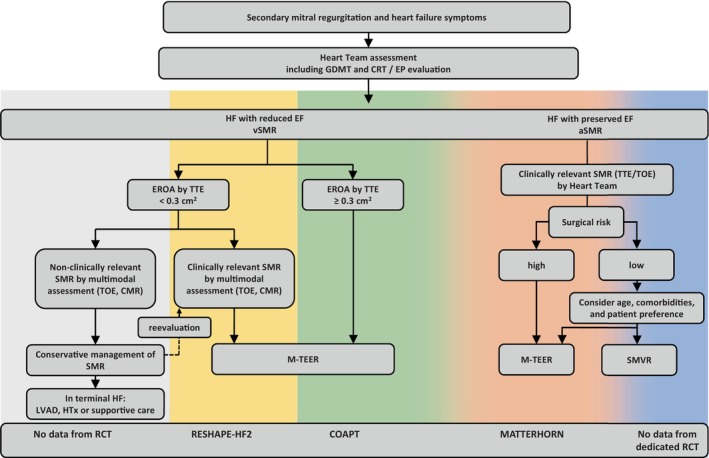
Proposed care pathway for patients with heart failure (HF) and secondary mitral regurgitation (SMR). aSMR, atrial secondary mitral regurgitation; CMR, cardiac magnetic resonance; CRT, cardiac resynchronization therapy; EF, ejection fraction; EP, electrophysiology; EROA, effective regurgitant orifice area; GDMT, guideline‐directed medical therapy; HTx, heart transplantation; LVAD, left ventricular assist device; M‐TEER, mitral valve transcatheter edge‐to‐edge repair; RCT, randomized controlled trial; SMVR, surgical mitral valve replacement; TOE, transoesophageal echocardiography; TTE, transthoracic echocardiography; vSMR, ventricular secondary mitral regurgitation.

Secondary mitral regurgitation is highly prevalent in the community and is associated with reduced quality of life and substantial risk of recurrent HFH and mortality. M‐TEER is effective in reducing symptoms and may improve outcomes in a larger subset of patients with symptomatic HFrEF than previously assumed. This includes patients with less severe MR and those at high risk for mitral valve surgery. Careful evaluation of MR pathology and LV size and function, implementation of optimal GDMT and CRT as well as exclusion of those with prognosis‐limiting comorbidities remain central perquisites to define those who will benefit from this therapy. *Figure* *2* summarizes evidence from randomized controlled studies for a proposed care pathway for patients with HF and SMR.

In aggregate, current data support earlier intervention in selected patients, and a reappraisal of the level of recomendation for this therapy in guidelines for treatment of HF in patients with SMR is warranted.

## References

[1] Lancellotti P , Pibarot P , Chambers J , La Canna G , Pepi M , Dulgheru R , *et al*. Multi‐modality imaging assessment of native valvular regurgitation: An EACVI and ESC Council of Valvular Heart Disease position paper. Eur Heart J Cardiovasc Imaging 2022;23:e171–e232. 10.1093/ehjci/jeab253 35292799

[2] Levine RA , Schwammenthal E . Ischemic mitral regurgitation on the threshold of a solution: From paradoxes to unifying concepts. Circulation 2005;112:745–758. 10.1161/CIRCULATIONAHA.104.486720 16061756

[3] Kumanohoso T , Otsuji Y , Yoshifuku S , Matsukida K , Koriyama C , Kisanuki A , *et al*. Mechanism of higher incidence of ischemic mitral regurgitation in patients with inferior myocardial infarction: Quantitative analysis of left ventricular and mitral valve geometry in 103 patients with prior myocardial infarction. J Thorac Cardiovasc Surg 2003;125:135–143. 10.1067/mtc.2003.78 12538997

[4] Yosefy C , Beeri R , Guerrero JL , Vaturi M , Scherrer‐Crosbie M , Handschumacher MD , *et al*. Mitral regurgitation after anteroapical myocardial infarction: New mechanistic insights. Circulation 2011;123:1529–1536. 10.1161/CIRCULATIONAHA.110.977843 21444880 PMC3085114

[5] Deferm S , Bertrand PB , Verbrugge FH , Verhaert D , Rega F , Thomas JD , *et al*. Atrial functional mitral regurgitation: JACC review topic of the week. J Am Coll Cardiol 2019;73:2465–2476. 10.1016/j.jacc.2019.02.061 31097168

[6] Silbiger JJ . Mechanistic insights into atrial functional mitral regurgitation: Far more complicated than just left atrial remodeling. Echocardiography 2019;36:164–169. 10.1111/echo.14249 30620100

[7] von Stein P , von Stein J , Hohmann C , Wienemann H , Guthoff H , Korber MI , *et al*. Atrial functional mitral regurgitation subtypes undergoing transcatheter edge‐to‐edge repair: Suboptimal outcomes in atriogenic hamstringing. JACC Cardiovasc Imaging 2025;18:16–29. 10.1016/j.jcmg.2024.06.019 39207336

[8] Dziadzko V , Clavel MA , Dziadzko M , Medina‐Inojosa JR , Michelena H , Maalouf J , *et al*. Outcome and undertreatment of mitral regurgitation: A community cohort study. Lancet 2018;391:960–969. 10.1016/S0140-6736(18)30473-2 29536860 PMC5907494

[9] Dziadzko V , Dziadzko M , Medina‐Inojosa JR , Benfari G , Michelena HI , Crestanello JA , *et al*. Causes and mechanisms of isolated mitral regurgitation in the community: Clinical context and outcome. Eur Heart J 2019;40:2194–2202. 10.1093/eurheartj/ehz314 31121021

[10] Chioncel O , Adamo M , Nikolaou M , Parissis J , Mebazaa A , Yilmaz MB , *et al*. Acute heart failure and valvular heart disease: A scientific statement of the Heart Failure Association, the Association for Acute CardioVascular Care and the European Association of Percutaneous Cardiovascular Interventions of the European Society of Cardiology. Eur J Heart Fail 2023;25:1025–1048. 10.1002/ejhf.2918 37312239

[11] Adamo M , Chioncel O , Benson L , Shahim B , Crespo‐Leiro MG , Anker SD , *et al*. Prevalence, clinical characteristics and outcomes of heart failure patients with or without isolated or combined mitral and tricuspid regurgitation: An analysis from the ESC‐HFA Heart Failure Long‐Term Registry. Eur J Heart Fail 2023;25:1061–1071. 10.1002/ejhf.2929 37365841

[12] Pagnesi M , Adamo M , Sama IE , Anker SD , Cleland JG , Dickstein K , *et al*. Clinical impact of changes in mitral regurgitation severity after medical therapy optimization in heart failure. Clin Res Cardiol 2022;111:912–923. 10.1007/s00392-022-01991-7 35294624 PMC9334376

[13] Pagnesi M , Adamo M , Sama IE , Anker SD , Cleland JG , Dickstein K , *et al*. Impact of mitral regurgitation in patients with worsening heart failure: Insights from BIOSTAT‐CHF. Eur J Heart Fail 2021;23:1750–1758. 10.1002/ejhf.2276 34164895 PMC9290728

[14] Pagnesi M , Adamo M , Ter Maaten JM , Beldhuis IE , Cotter G , Davison BA , *et al*. Impact of mitral regurgitation in patients with acute heart failure: Insights from the RELAX‐AHF‐2 trial. Eur J Heart Fail 2023;25:541–552. 10.1002/ejhf.2820 36915227

[15] Senni M , Adamo M , Metra M , Alfieri O , Vahanian A . Treatment of functional mitral regurgitation in chronic heart failure: Can we get a ‘proof of concept’ from the MITRA‐FR and COAPT trials? Eur J Heart Fail 2019;21:852–861. 10.1002/ejhf.1491 31116485

[16] Singh A , Su J , This A , Allaire S , Rouet JM , Laghi A , *et al*. A novel approach for semiautomated three‐dimensional quantification of mitral regurgitant volume reflects a more physiologic approach to mitral regurgitation. J Am Soc Echocardiogr 2022;35:940–946. 10.1016/j.echo.2022.05.005 35605896

[17] Conte C , Campoamor Cela C , Delgado V , Ferrer‐Sistach E . Atrial functional mitral regurgitation: The tip of the iceberg. Eur Heart J Cardiovasc Imaging 2024;25:e142. 10.1093/ehjci/jead303 37950880

[18] Lancellotti P , Troisfontaines P , Toussaint AC , Pierard LA . Prognostic importance of exercise‐induced changes in mitral regurgitation in patients with chronic ischemic left ventricular dysfunction. Circulation 2003;108:1713–1717. 10.1161/01.CIR.0000087599.49332.05 12975251

[19] Garg P , Swift AJ , Zhong L , Carlhall CJ , Ebbers T , Westenberg J , *et al*. Assessment of mitral valve regurgitation by cardiovascular magnetic resonance imaging. Nat Rev Cardiol 2020;17:298–312. 10.1038/s41569-019-0305-z 31819230 PMC7165127

[20] Cavalcante JL , Kusunose K , Obuchowski NA , Jellis C , Griffin BP , Flamm SD , *et al*. Prognostic impact of ischemic mitral regurgitation severity and myocardial infarct quantification by cardiovascular magnetic resonance. JACC Cardiovasc Imaging 2020;13:1489–1501. 10.1016/j.jcmg.2019.11.008 31864972

[21] Lopes BBC , Kwon DH , Shah DJ , Lesser JR , Bapat V , Enriquez‐Sarano M , *et al*. Importance of myocardial fibrosis in functional mitral regurgitation: From outcomes to decision‐making. JACC Cardiovasc Imaging 2021;14:867–878. 10.1016/j.jcmg.2020.10.027 33582069

[22] Arnold SV , Chinnakondepalli KM , Spertus JA , Magnuson EA , Baron SJ , Kar S , *et al*.; COAPT Investigators . Health status after transcatheter mitral‐valve repair in heart failure and secondary mitral regurgitation: COAPT trial. J Am Coll Cardiol 2019;73:2123–2132. 10.1016/j.jacc.2019.02.010 30894288 PMC6499691

[23] Januzzi JL , Omar AMS , Liu Y , Murphy S , Butler J , Felker GM , *et al*. Association between sacubitril/valsartan initiation and mitral regurgitation severity in heart failure with reduced ejection fraction: The PROVE‐HF study. Circulation 2022;146:1638–1640. 10.1161/CIRCULATIONAHA.122.061693 36183276 PMC9674443

[24] Kang DH , Park SJ , Shin SH , Hong GR , Lee S , Kim MS , *et al*. Angiotensin receptor neprilysin inhibitor for functional mitral regurgitation. Circulation 2019;139:1354–1365. 10.1161/CIRCULATIONAHA.118.037077 30586756

[25] Lowes BD , Gill EA , Abraham WT , Larrain JR , Robertson AD , Bristow MR , *et al*. Effects of carvedilol on left ventricular mass, chamber geometry, and mitral regurgitation in chronic heart failure. Am J Cardiol 1999;83:1201–1205. 10.1016/s0002-9149(99)00059-4 10215284

[26] Martens P , Belien H , Dupont M , Vandervoort P , Mullens W . The reverse remodeling response to sacubitril/valsartan therapy in heart failure with reduced ejection fraction. Cardiovasc Ther 2018;36:e12435. 10.1111/1755-5922.12435 29771478

[27] Nasser R , Van Assche L , Vorlat A , Vermeulen T , Van Craenenbroeck E , Conraads V , *et al*. Evolution of functional mitral regurgitation and prognosis in medically managed heart failure patients with reduced ejection fraction. JACC Heart Fail 2017;5:652–659. 10.1016/j.jchf.2017.06.015 28859754

[28] Waagstein F , Stromblad O , Andersson B , Bohm M , Darius M , Delius W , *et al*. Increased exercise ejection fraction and reversed remodeling after long‐term treatment with metoprolol in congestive heart failure: A randomized, stratified, double‐blind, placebo‐controlled trial in mild to moderate heart failure due to ischemic or idiopathic dilated cardiomyopathy. Eur J Heart Fail 2003;5:679–691. 10.1016/s1388-9842(03)00105-3 14607208

[29] Kang DH , Park SJ , Shin SH , Hwang IC , Yoon YE , Kim HK , *et al*. Ertugliflozin for functional mitral regurgitation associated with heart failure: EFFORT trial. Circulation 2024;149:1865–1874. 10.1161/CIRCULATIONAHA.124.069144 38690659

[30] Cleland JG , Daubert JC , Erdmann E , Freemantle N , Gras D , Kappenberger L , *et al*.; Cardiac Resynchronization‐Heart Failure (CARE‐HF) Study Investigators . The effect of cardiac resynchronization on morbidity and mortality in heart failure. N Engl J Med 2005;352:1539–1549. 10.1056/NEJMoa050496 15753115

[31] Ypenburg C , Lancellotti P , Tops LF , Bleeker GB , Holman ER , Pierard LA , *et al*. Acute effects of initiation and withdrawal of cardiac resynchronization therapy on papillary muscle dyssynchrony and mitral regurgitation. J Am Coll Cardiol 2007;50:2071–2077. 10.1016/j.jacc.2007.08.019 18021876

[32] Breithardt OA , Sinha AM , Schwammenthal E , Bidaoui N , Markus KU , Franke A , *et al*. Acute effects of cardiac resynchronization therapy on functional mitral regurgitation in advanced systolic heart failure. J Am Coll Cardiol 2003;41:765–770. 10.1016/s0735-1097(02)02937-6 12628720

[33] Verhaert D , Popovic ZB , De S , Puntawangkoon C , Wolski K , Wilkoff BL , *et al*. Impact of mitral regurgitation on reverse remodeling and outcome in patients undergoing cardiac resynchronization therapy. Circ Cardiovasc Imaging 2012;5:21–26. 10.1161/CIRCIMAGING.111.966580 22047983

[34] van der Bijl P , Khidir M , Ajmone Marsan N , Delgado V , Leon MB , Stone GW , *et al*. Effect of functional mitral regurgitation on outcome in patients receiving cardiac resynchronization therapy for heart failure. Am J Cardiol 2019;123:75–83. 10.1016/j.amjcard.2018.09.020 30539749

[35] Kawaji T , Shizuta S , Aizawa T , Yamagami S , Kato M , Yokomatsu T , *et al*. Impact of catheter ablation for atrial fibrillation on cardiac disorders in patients with coexisting heart failure. ESC Heart Fail 2021;8:670–679. 10.1002/ehf2.13160 33305495 PMC7835577

[36] Wu JT , Zaman JAB , Yakupoglu HY , Vennela B , Emily C , Nabeela K , *et al*. Catheter ablation of atrial fibrillation in patients with functional mitral regurgitation and left ventricular systolic dysfunction. Front Cardiovasc Med 2020;7:596491. 10.3389/fcvm.2020.596491 33381527 PMC7767831

[37] Adamo M , Fiorelli F , Melica B , D'Ortona R , Lupi L , Giannini C , *et al*. COAPT‐like profile predicts long‐term outcomes in patients with secondary mitral regurgitation undergoing MitraClip implantation. JACC Cardiovasc Interv 2021;14:15–25. 10.1016/j.jcin.2020.09.050 33309313

[38] Karam N , Stolz L , Orban M , Deseive S , Praz F , Kalbacher D , *et al*. Impact of right ventricular dysfunction on outcomes after transcatheter edge‐to‐edge repair for secondary mitral regurgitation. JACC Cardiovasc Imaging 2021;14:768–778. 10.1016/j.jcmg.2020.12.015 33582067

[39] Stolz L , Doldi PM , Orban M , Karam N , Puscas T , Wild MG , *et al*.; EuroSMR Investigators . Staging heart failure patients with secondary mitral regurgitation undergoing transcatheter edge‐to‐edge repair. JACC Cardiovasc Interv 2023;16:140–151. 10.1016/j.jcin.2022.10.032 36697148

[40] Tigges E , Blankenberg S , von Bardeleben RS , Zurn C , Bekeredjian R , Ouarrak T , *et al*. Implication of pulmonary hypertension in patients undergoing MitraClip therapy: Results from the German transcatheter mitral valve interventions (TRAMI) registry. Eur J Heart Fail 2018;20:585–594. 10.1002/ejhf.864 29575435

[41] Lindenfeld J , Abraham WT , Grayburn PA , Kar S , Asch FM , Lim DS , *et al*.; Cardiovascular Outcomes Assessment of the MitraClip Percutaneous Therapy for Heart Failure Patients With Functional Mitral Regurgitation (COAPT) Investigators . Association of effective regurgitation orifice area to left ventricular end‐diastolic volume ratio with transcatheter mitral valve repair outcomes: A secondary analysis of the COAPT trial. JAMA Cardiol 2021;6:427–436. 10.1001/jamacardio.2020.7200 33533873 PMC7859876

[42] Hausleiter J , Lachmann M , Stolz L , Bedogni F , Rubbio AP , Estevez‐Loureiro R , *et al*.; EuroSMR Investigators . Artificial intelligence‐derived risk score for mortality in secondary mitral regurgitation treated by transcatheter edge‐to‐edge repair: The EuroSMR risk score. Eur Heart J 2024;45:922–936. 10.1093/eurheartj/ehad871 38243773

[43] Adamo M , Tomasoni D , Stolz L , Stocker TJ , Pancaldi E , Koell B , *et al*. Impact of transcatheter edge‐to‐edge mitral valve repair on guideline‐directed medical therapy uptitration. JACC Cardiovasc Interv 2023;16:896–905. 10.1016/j.jcin.2023.01.362 37100553

[44] Lindman BR , Asch FM , Grayburn PA , Mack MJ , Bax JJ , Gonzales H , *et al*. Ventricular remodeling and outcomes after mitral transcatheter edge‐to‐edge repair in heart failure: The COAPT trial. JACC Cardiovasc Interv 2023;16:1160–1172. 10.1016/j.jcin.2023.02.031 37225286

[45] Hausleiter J , Stocker TJ , Adamo M , Karam N , Swaans MJ , Praz F . Mitral valve transcatheter edge‐to‐edge repair. EuroIntervention 2023;18:957–976. 10.4244/EIJ-D-22-00725 36688459 PMC9869401

[46] Kassar M , Praz F , Hunziker L , Pilgrim T , Windecker S , Seiler C , *et al*. Anatomical and technical predictors of three‐dimensional mitral valve area reduction after transcatheter edge‐to‐edge repair. J Am Soc Echocardiogr 2022;35:96–104. 10.1016/j.echo.2021.08.021 34506920

[47] Stone GW , Lindenfeld J , Abraham WT , Kar S , Lim DS , Mishell JM , *et al*.; COAPT Investigators . Transcatheter mitral‐valve repair in patients with heart failure. N Engl J Med 2018;379:2307–2318. 10.1056/NEJMoa1806640 30280640

[48] Sugiura A , Kavsur R , Spieker M , Iliadis C , Goto T , Ozturk C , *et al*. Recurrent mitral regurgitation after MitraClip: Predictive factors, morphology, and clinical implication. Circ Cardiovasc Interv 2022;15:e010895. 10.1161/CIRCINTERVENTIONS.121.010895 35193380

[49] Tabata N , Weber M , Sugiura A , Ozturk C , Ishii M , Tsujita K , *et al*. Impact of the leaflet‐to‐annulus index on residual mitral regurgitation in patients undergoing edge‐to‐edge mitral repair. JACC Cardiovasc Interv 2019;12:2462–2472. 10.1016/j.jcin.2019.09.014 31857016

[50] Tanaka T , Sugiura A , Ozturk C , Vogelhuber J , Tabata N , Wilde N , *et al*. Transcatheter edge‐to‐edge repair for atrial secondary mitral regurgitation. JACC Cardiovasc Interv 2022;15:1731–1740. 10.1016/j.jcin.2022.06.005 36075644

[51] von Bardeleben RS , Rogers JH , Mahoney P , Price MJ , Denti P , Maisano F , *et al*. Real‐world outcomes of fourth‐generation mitral transcatheter repair: 30‐day results from EXPAND G4. JACC Cardiovasc Interv 2023;16:1463–1473. 10.1016/j.jcin.2023.05.013 37380228

[52] von Stein P , Stolz L , Haurand JM , Groger M , Rudolph F , Mustafa D , *et al*. Outcomes and impact of device iterations in mitral valve transcatheter edge‐to‐edge repair: The REPAIR study. JACC Cardiovasc Interv 2025;18:573–586. 10.1016/j.jcin.2024.11.016 39745410

[53] Hell MM , Wild MG , Baldus S , Rudolph T , Treede H , Petronio AS , *et al*.; TENDER Investigators . Transapical mitral valve replacement: 1‐year results of the real‐world Tendyne European Experience registry. JACC Cardiovasc Interv 2024;17:648–661. 10.1016/j.jcin.2023.12.027 38385922

[54] McDonagh TA , Metra M , Adamo M , Gardner RS , Baumbach A , Böhm M , *et al*. 2021 ESC Guidelines for the diagnosis and treatment of acute and chronic heart failure: Developed by the Task Force for the diagnosis and treatment of acute and chronic heart failure of the European Society of Cardiology (ESC). With the special contribution of the Heart Failure Association (HFA) of the ESC. Eur J Heart Fail 2022;24:4–131. 10.1002/ejhf.2333 35083827

[55] Heidenreich PA , Bozkurt B , Aguilar D , Allen LA , Byun JJ , Colvin MM , *et al*. 2022 AHA/ACC/HFSA guideline for the management of heart failure: Executive summary: A report of the American College of Cardiology/American Heart Association Joint Committee on Clinical Practice Guidelines. J Am Coll Cardiol 2022;79:1757–1780. 10.1016/j.jacc.2021.12.011 35379504

[56] Otto CM , Nishimura RA , Bonow RO , Carabello BA , Erwin JP 3rd , Gentile F , *et al*. 2020 ACC/AHA guideline for the management of patients with valvular heart disease: Executive summary: A report of the American College of Cardiology/American Heart Association Joint Committee on Clinical Practice Guidelines. J Am Coll Cardiol 2021;77:450–500. 10.1016/j.jacc.2020.11.035 33342587

[57] Praz F , Borger MA , Lanz J , Marin‐Cuartas M , Abreu A , Adamo M , *et al*.; ESC/EACTS Scientific Document Group . 2025 ESC/EACTS Guidelines for the management of valvular heart disease. Eur Heart J 2025:ehaf194. 10.1093/eurheartj/ehaf194

[58] Anker SD , Friede T , von Bardeleben RS , Butler J , Khan MS , Diek M , *et al*.; RESHAPE‐HF2 Investigators . Transcatheter valve repair in heart failure with moderate to severe mitral regurgitation. N Engl J Med 2024;391:1799–1809. 10.1056/NEJMoa2314328 39216092

[59] Obadia JF , Messika‐Zeitoun D , Leurent G , Iung B , Bonnet G , Piriou N , *et al*.; MITRA‐FR Investigators . Percutaneous repair or medical treatment for secondary mitral regurgitation. N Engl J Med 2018;379:2297–2306. 10.1056/NEJMoa1805374 30145927

[60] Anker MS , Porthun J , Bonnet G , Schulze PC , Rassaf T , Landmesser U . Percutaneous transcatheter edge‐to‐edge repair for functional mitral regurgitation in heart failure: A meta‐analysis of 3 randomized controlled trials. J Am Coll Cardiol 2024;84:2364–2368. 10.1016/j.jacc.2024.08.026 39217568

[61] Anker SD , Friede T , von Bardeleben RS , Butler J , Fatima K , Diek M , *et al*. Randomized investigation of the MitraClip device in heart failure: Design and rationale of the RESHAPE‐HF2 trial design. Eur J Heart Fail 2024;26:984–993. 10.1002/ejhf.3247 38654139

[62] Anker SD , Friede T , von Bardeleben RS , Butler J , Khan MS , Diek M , *et al*. Percutaneous repair of moderate‐to‐severe or severe functional mitral regurgitation in patients with symptomatic heart failure: Baseline characteristics of patients in the RESHAPE‐HF2 trial and comparison to COAPT and MITRA‐FR trials. Eur J Heart Fail 2024;26:1608–1615. 10.1002/ejhf.3286 38847420

[63] Grayburn PA , Sannino A , Packer M . Proportionate and disproportionate functional mitral regurgitation: A new conceptual framework that reconciles the results of the MITRA‐FR and COAPT trials. JACC Cardiovasc Imaging 2019;12:353–362. 10.1016/j.jcmg.2018.11.006 30553663

[64] Ponikowski P , Friede T , von Bardeleben RS , Butler J , Shahzeb Khan M , Diek M , *et al*. Hospitalization of symptomatic patients with heart failure and moderate to severe functional mitral regurgitation treated with MitraClip: Insights from RESHAPE‐HF2. J Am Coll Cardiol 2024;84:2347–2363. 10.1016/j.jacc.2024.08.027 39217574

[65] Lurz P , Schmitz T , Geisler T , Hausleiter J , Eitel I , Rudolph V , *et al*.; MiCLASP Study Investigators . Mitral valve transcatheter edge‐to‐edge repair: 1‐year outcomes from the MiCLASP study. JACC Cardiovasc Interv 2024;17:890–903. 10.1016/j.jcin.2024.02.022 38599692

[66] Asgar AW , Tang GHL , Rogers JH , Rottbauer W , Morse MA , Denti P , *et al*. Evaluating mitral TEER in the management of moderate secondary mitral regurgitation among heart failure patients. JACC Heart Fail 2025;13:213–225. 10.1016/j.jchf.2024.08.001 39269396

[67] Gupta A , Packer M , Makkar R , Grayburn P . A volume‐based framework reconciling COAPT, MITRA‐FR, and RESHAPE‐HF2. J Am Coll Cardiol 2024;84:2376–2379. 10.1016/j.jacc.2024.08.029 39320294

[68] Stolz L , Stocker TJ , Lurz P , Hausleiter J . Growing evidence for edge‐to‐edge repair in secondary mitral regurgitation: What to learn from COAPT, MITRA‐FR, and RESHAPE‐HF2. JACC Cardiovasc Interv 2025;18:927–932. 10.1016/j.jcin.2025.01.429 40240087

[69] Acker MA , Parides MK , Perrault LP , Moskowitz AJ , Gelijns AC , Voisine P , *et al*.; CTSN . Mitral‐valve repair versus replacement for severe ischemic mitral regurgitation. N Engl J Med 2014;370:23–32. 10.1056/NEJMoa1312808 24245543 PMC4128011

[70] Goldstein D , Moskowitz AJ , Gelijns AC , Ailawadi G , Parides MK , Perrault LP , *et al*.; CTSN . Two‐year outcomes of surgical treatment of severe ischemic mitral regurgitation. N Engl J Med 2016;374:344–353. 10.1056/NEJMoa1512913 26550689 PMC4908819

[71] Nappi F , Lusini M , Spadaccio C , Nenna A , Covino E , Acar C , *et al*. Papillary muscle approximation versus restrictive annuloplasty alone for severe ischemic mitral regurgitation. J Am Coll Cardiol 2016;67:2334–2346. 10.1016/j.jacc.2016.03.478 27199056

[72] Pausch J , Harmel E , Sinning C , Reichenspurner H , Girdauskas E . Standardized subannular repair for type IIIb functional mitral regurgitation in a minimally invasive mitral valve surgery settingdagger. Eur J Cardiothorac Surg 2019;56:968–975. 10.1093/ejcts/ezz114 31005995

[73] Baldus S , Doenst T , Pfister R , Gummert J , Kessler M , Boekstegers P , *et al*.; MATTERHORN Investigators . Transcatheter repair versus mitral‐valve surgery for secondary mitral regurgitation. N Engl J Med 2024;391:1787–1798. 10.1056/NEJMoa2408739 39216093

[74] Rudolph F , Geyer M , Baldus S , De Luca VM , Doenst T , Pfister R , *et al*. Transcatheter repair versus surgery for atrial versus ventricular functional mitral regurgitation: A post hoc analysis of the MATTERHORN trial. Circulation 2025;151:418–420. 10.1161/CIRCULATIONAHA.124.072648 39475706

[75] Mann DL , Givertz MM , Vader JM , Starling RC , Shah P , McNulty SE , *et al*.; LIFE Investigators . Effect of treatment with sacubitril/valsartan in patients with advanced heart failure and reduced ejection fraction: A randomized clinical trial. JAMA Cardiol 2022;7:17–25. 10.1001/jamacardio.2021.4567 34730769 PMC8567189

[76] Cox ZL , Zalawadiya SK , Simonato M , Redfors B , Zhou Z , Kotinkaduwa L , *et al*. Guideline‐directed medical therapy tolerability in patients with heart failure and mitral regurgitation: The COAPT trial. JACC Heart Fail 2023;11:791–805. 10.1016/j.jchf.2023.03.009 37115135

[77] Mullens W , Dauw J , Gustafsson F , Mebazaa A , Steffel J , Witte KK , *et al*. Integration of implantable device therapy in patients with heart failure. A clinical consensus statement from the Heart Failure Association (HFA) and European Heart Rhythm Association (EHRA) of the European Society of Cardiology (ESC). Eur J Heart Fail 2024;26:483–501. 10.1002/ejhf.3150 38269474

[78] Leyva F , Zegard A , Patel P , Stegemann B , Marshall H , Ludman P , *et al*. Timing of cardiac resynchronization therapy implantation. Europace 2023;25:euad059. 10.1093/europace/euad059 36944529 PMC10227865

[79] Marrouche NF , Brachmann J , Andresen D , Siebels J , Boersma L , Jordaens L , *et al*.; CASTLE‐AF Investigators . Catheter ablation for atrial fibrillation with heart failure. N Engl J Med 2018;378:417–427. 10.1056/NEJMoa1707855 29385358

[80] Sohns C , Fox H , Marrouche NF , Crijns H , Costard‐Jaeckle A , Bergau L , *et al*.; CASTLE HTx Investigators . Catheter ablation in end‐stage heart failure with atrial fibrillation. N Engl J Med 2023;389:1380–1389. 10.1056/NEJMoa2306037 37634135

[81] Gertz ZM , Raina A , Mountantonakis SE , Zado ES , Callans DJ , Marchlinski FE , *et al*. The impact of mitral regurgitation on patients undergoing catheter ablation of atrial fibrillation. Europace 2011;13:1127–1132. 10.1093/europace/eur098 21490035

[82] Masuda M , Sekiya K , Asai M , Iida O , Okamoto S , Ishihara T , *et al*. Influence of catheter ablation for atrial fibrillation on atrial and ventricular functional mitral regurgitation. ESC Heart Fail 2022;9:1901–1913. 10.1002/ehf2.13896 35293159 PMC9065851

[83] Gertz ZM , Herrmann HC , Lim DS , Kar S , Kapadia SR , Reed GW , *et al*. Implications of atrial fibrillation on the mechanisms of mitral regurgitation and response to MitraClip in the COAPT trial. Circ Cardiovasc Interv 2021;14:e010300. 10.1161/CIRCINTERVENTIONS.120.010300 33719505

[84] Haberman D , Estevez‐Loureiro R , Czarnecki A , Melillo F , Adamo M , Villablanca P , *et al*. Transcatheter edge‐to‐edge repair in severe mitral regurgitation following acute myocardial infarction – aetiology‐based analysis. Eur J Heart Fail 2025;27:912–921. 10.1002/ejhf.3582 39809715

[85] Dimitriadis K , Soulaidopoulos S , Pyrpyris N , Sagris M , Aznaouridis K , Beneki E , *et al*. Transcatheter edge‐to‐edge repair for severe mitral regurgitation in patients with cardiogenic shock: A systematic review and meta‐analysis. J Am Heart Assoc 2025;14:e034932. 10.1161/JAHA.124.034932 40055145 PMC12132604

[86] Stone GW , Abraham WT , Lindenfeld J , Kar S , Grayburn PA , Lim DS , *et al*.; COAPT Investigators . Five‐year follow‐up after transcatheter repair of secondary mitral regurgitation. N Engl J Med 2023;388:2037–2048. 10.1056/NEJMoa2300213 36876756

